# Pharmacist-driven outreach initiative to increase prescribing of sodium-glucose cotransporter-2 inhibitors in eligible VHA patients with chronic kidney disease: a study protocol

**DOI:** 10.1186/s12882-023-03446-1

**Published:** 2024-01-05

**Authors:** Deborah L. Pestka, Daniel Murphy, Pearl Huynh, Jessica A. Rechtzigel, Shari Kjos, Lisa Marie Ellich, Adam N. Kaplan, Brent C. Taylor, Melissa Atwood, Beth A. Polsfuss, Joseph Y. Lee, Areef Ishani

**Affiliations:** 1 Department of Medicine, University of Minnesota, Minneapolis, MN USA; 2 Minneapolis VA Health Care System, 1 Veterans Drive, Minneapolis, MN 55417 USA

**Keywords:** Chronic Kidney Disease, Implementation strategy, Clinical pharmacy services

## Abstract

**Background:**

Patients with chronic kidney disease (CKD) are at increased risk for multiple adverse events, several of which have been proven to be less likely with the use of sodium-glucose cotransporter-2 inhibitors (SGLT2i). As a result, guidelines now recommend SGLT2i be given to those with mild to moderate CKD and type 2 diabetes. The objective of this study is to evaluate if a pharmacist-driven SGLT2i prescribing initiative among eligible patients with CKD and diabetes within the VA could more rapidly improve the adoption of SGLT2i via a pragmatic approach aligned with learning health systems.

**Methods:**

Eligible patients will be identified through an established VA diabetes dashboard. Veterans with an odd social security number (SSN), which is effectively a random number, will be the intervention group. Those with even SSNs will serve as the control while awaiting a second iteration of the same interventional program. The intervention will be implemented in a rolling fashion across one Veterans Integrated Service Network. Our primary outcome is initiation of an SGLT2i. Secondary outcomes will include medication adherence and safety-related outcomes.

**Discussion:**

This project tests the impact of a pharmacist-driven medication outreach initiative as a strategy to accelerate initiation of SGLT2i. The results of this work will not only illustrate the effectiveness of this strategy for SGLT2is but may also have implications for increasing other guideline-concordant care. Furthermore, the utilization of SSNs to select Veterans for the first wave of this program has created a pseudo-randomized interventional trial supporting a pragmatic learning health system approach.

**Trial registration:**

ISRCTN12374636.

**Supplementary Information:**

The online version contains supplementary material available at 10.1186/s12882-023-03446-1.

## Background

Chronic kidney disease (CKD) is a common condition affecting more than 1 in 7, or approximately 15%, of U.S. adults [1]. Through various mechanisms, patients with kidney disease are known to be at heightened risk for cardiovascular disease and cardiovascular events [2, 3]. However, several recent studies have been published documenting the benefit of sodium-glucose co-transporter 2 inhibitors (SGLT2i) on reducing cardiovascular death and major adverse cardiovascular outcomes in patients with CKD, as well as decreasing the development or worsening of nephropathy [4–11]. SGLT2i are a class of drugs approved for the treatment of type 2 diabetes. Given the emerging research that has been produced on the benefits of SGLT2i, the American Diabetes Association (ADA) and the Kidney Disease Improving Global Outcomes (KDIGO) guidelines now recommend that SGLT2i be given to all patients with mild to moderate CKD and type 2 diabetes regardless of their glycemic control [12, 13]. Nevertheless, prescribing SGLT2i for eligible patients remains suboptimal [14–16]. Previous research has indicated that it can take as much as 17 years for evidence to become solidified into routine practice and care [17]. Therefore, given the newfound benefits of SGLT2i in patients with CKD and type 2 diabetes and recent changes to treatment guidelines, what remains unknown is how to effectively implement these guidelines into practice to improve the health of patients with CKD.

Pharmacists play a key role in patient care teams. Numerous studies have demonstrated that integrating pharmacists into the care team results in improved clinical outcomes, decreased health care costs, improved patient experience, and improved care team experience – all aspects of the quintessential Quadruple Aim [18–23]. In addition, pharmacists can prescribe medications in many states, improving their ability to optimize and manage medications. Given their expertise in medication management and the success of other pharmacist-focused interventions, [24–26] an implementation strategy for increasing SGLT2i that incorporates clinical pharmacists holds great promise.

Testing and evaluating implementation strategies, such as pharmacist-driven SGLT2i proactive outreach, also supports and aligns with the principles of learning health systems. A learning health system is one in which internal data and experience are integrated with external evidence to produce knowledge that can be used to improve practice [27]. While the effectiveness of SGLT2i have been well documented, strategies to improve prescribing of this class of medication to eligible patients are needed. In alignment with learning health systems, it is also important that the evaluation of these strategies remain pragmatic in nature so that they can be easily tested, yet maintain scientific rigor to adequately determine effectiveness.

### Aims

Improve the proportion of eligible Veterans with CKD initiated on an SGLT2i through a targeted pharmacy intervention across a series of Veterans Affairs (VA) clinics. In addition, this project aims to inform learning health systems by testing a pragmatic strategy to reach eligible patients.

We hypothesize that patients who receive a targeted pharmacy intervention during the first iteration will have a higher proportion of SGLT2i initiation than those receiving usual care while they await a future wave of this same interventional program.

## Methods

### Study design and setting

The VA is divided into different regional systems of care across the U.S. called Veterans Integrated Service Networks (VISNs). This multicenter interventional study focused on the Midwest VISN, which includes 8 VA health systems across Minnesota, Iowa, North Dakota, South Dakota, and Nebraska. Eligible Veterans are assigned to either a targeted pharmacy intervention or usual care. The study began in February 2022 and will continue until all eligible Veterans in the region are offered the intervention, which is anticipated to be in 2025.

### Ethical and regulatory approval

The Minneapolis VA Health Care System Human Research Protection Program determined that, as a quality improvement initiative, this study did not meet the definition of research and, therefore, did not need institutional review board (IRB) oversight. This determination extends to all VA sites as they are all within the same health system.

### Eligibility criteria

Patients are included in the study if they have the following inclusion criteria:

- CKD (defined as estimated glomerular filtration rate (eGFR), as extracted from the VA electronic health record, ≥ 25 mL/min/1.73 m^2^ and eGFR < 60 mL/min/1.73 m^2^ upon two measurements).
- Type 2 diabetes (defined as either ICD9/10 code of type 2 diabetes or most recent hemoglobin A1c > = 7%).

Patients are excluded if they:

- Have type 1 diabetes.
- Are already prescribed an SGLT2i or GLP-1 medication at the time of data acquisition, which serves as the index date.
- Have an SGLT2i allergy.
- Are on dialysis.
- Have a diagnosis of pancreatic cancer or pancreatitis.

### Patient selection and randomization

As part of their Academic Detailing Diabetes Campaign and to monitor SGLT2i initiation, the VA developed a diabetes dashboard to identify patients who would benefit from an SGLT2i. The dashboard includes the above inclusion criteria and several other demographic and clinical variables. The dashboard is updated daily to reflect eligible patients. One included variable in the dashboard is patients’ social security number. Due to the finite availability of resources, we have utilized a pseudo-randomization of eligible Veterans to determine which Veterans within the VISN receive the intervention first, providing a natural comparison group in those Veterans who will be intervened upon later. For this study, patients with an odd social security number are put in the intervention arm, while those with an even social security number serve as the control. Patient lists will be extracted from the VA dashboard at fixed time points throughout the study and will be considered the dates of randomization. The controls will receive usual care until all eligible Veterans in the odd-numbered group have received the intervention. At that time, even-numbered Veterans will also begin to receive the intervention.

The intervention will be implemented in a rolling fashion, beginning with patients at the Minneapolis VA health system, and other clinics will be subsequently added as outreach is being completed.

### Intervention

**Fig. 1:**
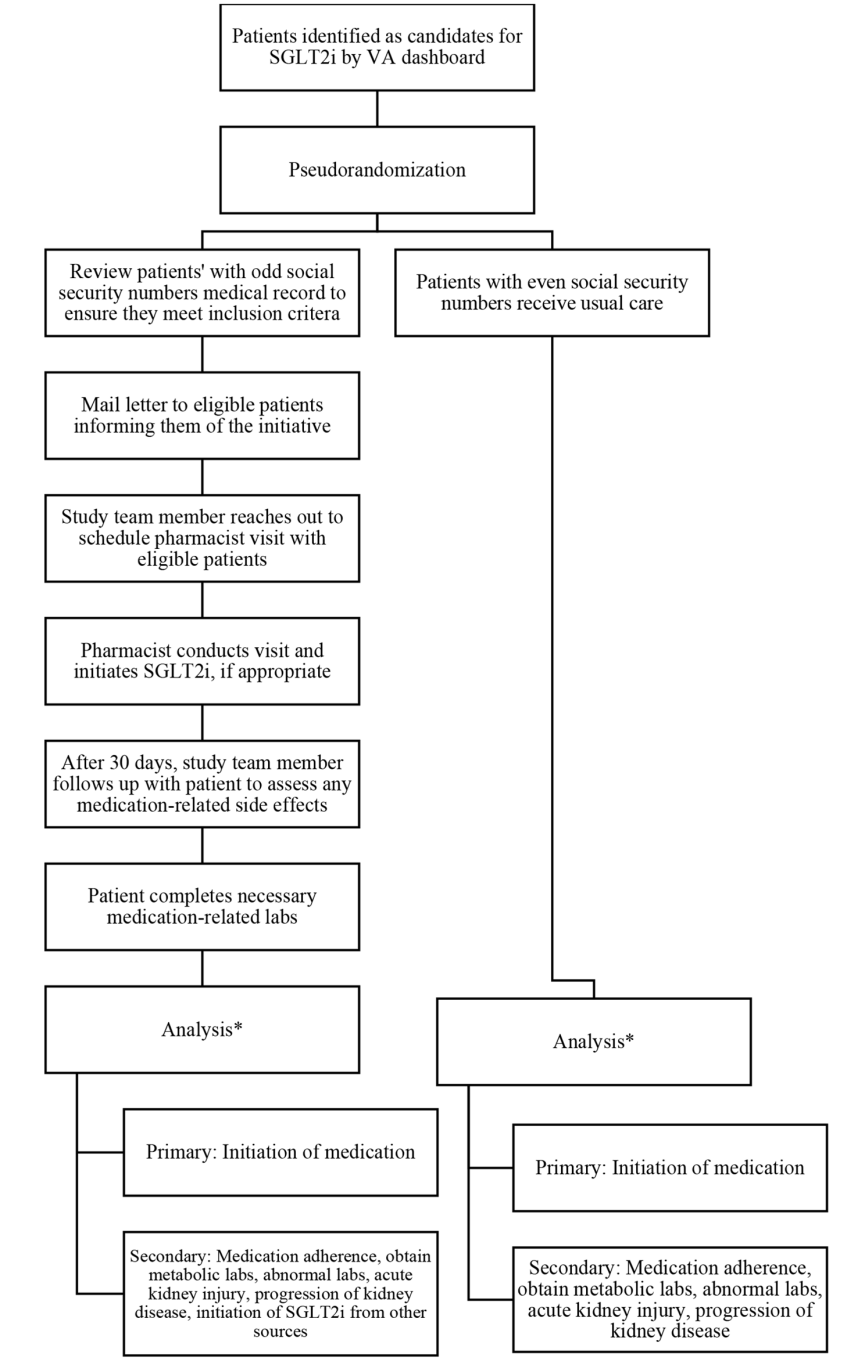
Care processes and analysis of study arms. ^*^Analyses will include all patients identified in the VA dashboard

The intervention steps are illustrated in Fig. 1. Because the patient lists are pulled at fixed time points, which serve as an index date, a patient may have been prescribed an SGLT2i between when the list is generated and when intervention outreach occurs. For that reason, the first step of the process is that a study team member reviews the patient’s medical record to ensure that they have not already been prescribed the medication and that they meet all other inclusion criteria. However, if a patient is prescribed a GLP-1 between their index date and their date of pharmacist review, they are not subsequently excluded. If patients otherwise continue to meet the study criteria, they are mailed a letter describing the benefits of SGLT2i, informed of the initiative, and instructed that they will receive a call to set up an appointment with the clinical pharmacist. After patients have received their letter, a member of the study team calls them to request their participation and to schedule a visit with the clinical pharmacist. Patients who agree are seen by one of two clinical pharmacists dedicated to this project. Pharmacist appointments are scheduled for 30 minutes, during which the pharmacist assesses the patient’s current medications and disease states that may be impacted by an SGLT2i, such as self-monitored blood glucose levels, perceived life expectancy, and any other relevant information. During this time, the pharmacist also addresses any questions or concerns the patient may have about the medication. If the pharmacist determines that a patient is an ideal candidate for the medication and the patient agrees, empagliflozin will be prescribed to the patient.

Approximately 30 days after starting the medication, a member of the study team calls the patient to assess for medication side effects. If the patient tolerates the medication, follow-up labs will be scheduled to reassess the patient’s basic metabolic panel. However, if the patient has side effects or concerns, the patient will receive a follow-up phone call and assessment by the pharmacist to determine if empagliflozin should be continued. If the one-month lab results come back abnormal, the patient will have a follow-up outreach to determine the appropriate course of action and to engage with the patient’s primary care team. If the lab results are stable and within normal limits, the patient will receive a letter with their lab results and continued care from their primary care team. Patients in the control arm will receive usual care while the first iteration of this program is ongoing and may be prescribed an SGLT2i, if recommended by their care team.

### Clinical team

There are two full-time equivalent (FTE) clinical pharmacists to carry out the intervention. In addition, to support patient outreach and follow-up, the team also includes one FTE for a medical assistant and one FTE for a licensed practical nurse (LPN).

### Outcomes

Our primary outcome is initiation of an SGLT2i among patients deemed candidates for SGLT2i as determined by the VA dashboard. Patients who filled an SGLT2i at any point during the study timeframe, as determined by electronic health record data, will be considered to have initiated the medication.

Secondary outcomes will include adherence to the medication 12 months after initial prescribing (defined by refills of their SGLT2i), as well as various process and safety measures, such as the number and percent of patients who obtained metabolic labs after SGLT2i initiation, abnormal lab results, including acute kidney injury, and defined reasons for declining or stopping the medication (including low blood pressure/hypotension and hypoglycemia). Secondary outcomes will also include the proportion of patients who decline the intervention and later obtain an SGLT2i from another source (which will be reported as a descriptive statistic only as Veterans in the usual care, unlike those in the intervention arm, cannot crossover to the alternative arm). In addition, we will examine the progression of kidney disease, defined as a 40% reduction in eGFR sustained for at least two measures. We will also measure the number of times eGFR is assessed as this may affect outcome ascertainment.

Exploratory outcomes will include rates of all-cause hospitalizations, initiation of dialysis, and major adverse cardiac events (nonfatal myocardial infarction, nonfatal stroke, and hospitalization for heart failure).

### Data collection

All process data are collected through a tracking app developed specifically for this project. Clinical outcome data are collected and stored in the VA electronic health record.

### Analysis

We will assess balance in baseline clinical and demographic characteristics between the two study arms with chi-square tests for categorical variables (e.g., patient sex) and Kruskal-Wallis test assuming unequal variances for continuous variables (e.g., patient age, baseline eGFR as calculated by the final serum creatinine concentration before the index date via the most recent CKD-EPI equation) [28]. The primary and secondary outcomes, the proportion of initiation of SGLT2i or proportion with a safety or process measure, respectively, will be compared between the study arms via the Two-Sample Proportions test. For binary exploratory outcomes, such as initiation of dialysis, major adverse cardiac events, and progression of kidney disease, we will also compare proportions between the arms via Two-Sample Proportions test. When these binary outcomes have an associated time of occurrence, Kaplan-Meier analysis will be used to assess the cumulative incidence of these events. We will use logistic regression and Cox proportional hazards models for adjusted analyses accounting for patient medical and demographic characteristics for binary and time-till-event outcomes. A Type-I-Error rate of 0.05 will be used for the primary outcome to declare significance and we will report the associated 95% confidence interval. Secondary outcomes will also use a p-value of < 0.05 and 95% confidence intervals.

## Discussion

Given the emergent evidence on the benefits of SGLT2i on cardiac and kidney health, initiating eligible patients on this medication is of great importance and aligns with current practice guidelines. However, changing clinician prescribing behavior is a challenge; [29, 30] therefore, relying on passive diffusion by waiting for clinicians to change their prescribing patterns may take significant time. To expedite the implementation of SGLT2i prescribing, implementation strategies that promote adoption and penetration are needed. Due to the favorable risk profile of SGLT2i [5, 7] and the availability of local laboratory testing as routine post-SGLT2i assessment, this medication class yields itself well to both pharmacist-led care and a time-limited intervention before returning to primary care.

This project tests the effectiveness of a pharmacist-driven medication outreach initiative as a strategy to accelerate the initiation of SGLT2i in a rolling fashion to eligible patients within the VA. It will also provide data on the frequency with which patients reject SGLT2i, either when offered or due to early side effects or concerns after initiation outside the confines of a randomized clinical trial. If deemed successful, such a project would also offer scalability in both the VA and other health systems utilizing clinical pharmacy services. In addition, the results of this work will not only illustrate the effectiveness of this strategy in initiating SGLT2i but may also have implications for increasing dissemination for other areas of guideline-concordant care.

Finally, this design method lends itself well to the principles of learning health systems. Having systems in place to rapidly generate evidence and support practice change are key to the continuous learning and improvement that occurs within a learning health system. The rolling nature of this initiative and our election to use social security number, which is effectively a randomly assigned number (in the final digit) at birth, to create an intervention arm has created a pseudo-randomized interventional trial. Similar pragmatic approaches should be explored with future quality improvement projects to facilitate study designs that are easily implementable yet have added rigor to produce generalizable findings.

### Electronic supplementary material

Below is the link to the electronic supplementary material.

Supplementary Material 1

## Data Availability

No datasets were generated or analysed during the current study.

## Data Availability

Not applicable for study protocol.

## Abbreviations

CKD: Chronic kidney disease
SGLT2i: Sodium glucose co-transport 2 inhibitor
ADA: American Diabetes Association
VA: Veterans Affairs
VISN: Veterans Integrated Service Networks
ICD: International classification of diseases
A1c: Hemoglobin A1c
GLP: Glucagon-like peptide
FTE: Full-time equivalent
LPN: Licensed practical nurse
eGFR: Estimated glomerular filtration
