# Supplementary material for: Pharmacist-driven outreach initiative to increase prescribing of sodium-glucose cotransporter-2 inhibitors in eligible VHA patients with chronic kidney disease: a study protocol
Source: BMC Nephrol. 2024 Jan 5;25:14. doi: 10.1186/s12882-023-03446-1 (PMC10770983; doi:10.1186/s12882-023-03446-1)
Supplement: Supplementary file 1 — Supplementary Material 1 [file 12882_2023_3446_MOESM1_ESM.pdf]

Department of Veterans Affairs

Memorandum

Date: January 26, 2022

To: Areef Ishani, MD, MS

From: HRPP Director, Minneapolis VA Health Care System

Re: Project: ***SGLT2i to prevent cardiovascular disease and dialysis in patients with chronic kidney disease (CKD)***

Cc: Larry Brown, MD – VISN 23 CMO

1. The engagement on the part of the Minneapolis VA Health Care System as part of the referenced project has been reviewed and determined to not meet the definition of research. 45 CFR 46 102(l) defines research as "*a systematic investigation, including research development, testing and evaluation, designed to develop or contribute to generalizable knowledge*". Generalizable knowledge, as defined by VA Program Guide 1200.21 §4.a., is "*information that expands the knowledge base of a scientific discipline or other scholarly field of study*".
2. Oversight by the IRB or other Research oversight committees *is not* required. Please be aware that for this project you do need to follow all VA privacy and data security rules governing clinical practice.
3. Research Service does not assume any responsibility for tracking or monitoring compliance with any VA guidance governing the conduct, publication or presentation requirements of non-research (i.e., operations) activities. For more information on this guidance consult VA Program Guide 1200.21.
4. Should anything in your project design change which may affect this non-research determination, please contact IRBMN@VA.GOV prior to implementing the change.
5. Please do not hesitate to contact IRBMN@VA.GOV if there are questions or concerns regarding this determination.

Julie A. Toth 414232

Digitally signed by Julie A. Toth 414232
Date: 2022.01.26 15:38:13 -06'00'

Title: SGLT2i to prevent cardiovascular disease and dialysis in patients with chronic kidney disease (CKD)

Purpose

Recent studies have shown that when patients with chronic kidney disease take a class of medication for diabetes, sodium glucose co-transport 2 inhibitors (SGLT2i), they have reduced rates of major adverse cardiovascular outcomes, cardiovascular death, and decreased incidence of developing or worsening nephropathy. Despite documented benefits, the current process for disseminating this treatment has only resulted in 6-8% of eligible VA patients on therapy. These drugs are not limited currently and can be prescribed by any VA provider.

Funded by a VISN 23 Strategic Initiative, we propose an implementation plan initially at the Minneapolis and St. Cloud VA Medical Centers, ultimately expanding to all health care systems in VISN 23, to improve SGLT2i treatment rates among Veterans with chronic kidney disease and type 2 diabetes. We plan to evaluate implementation and effectiveness outcomes of initiating a pharmacy service in which pharmacists will meet with eligible patients with chronic kidney disease to review their medical history and initiate them on SGLT2i therapy, if appropriate. The goal of this internal VA evaluation is to assess the implementation benefits of the pharmacy service program, which if found to have demonstrated benefits will be subsequently rolled out to the rest of VISN 23 and the nation.

Procedures

Using the established VA academic detailing dashboard that identifies chronic kidney disease patients that are candidates for SGLT2i therapy, we plan to send half of eligible patients a letter informing them of the benefits of this medication (the letter will be sent to patients whose social security number ends with an odd digit). A medical assistant will then reach out to those patients and offer to schedule a consultation with one of our two intervention pharmacists. The pharmacist will review the patients' charts and ensure they meet necessary criteria (e.g., diagnosis of chronic kidney disease, diagnosis of type 2 diabetes, eGFR > 25 mL/min) for initiating the drug and no exclusion criteria (e.g., type 1 diabetes, history of diabetic ketoacidosis) exist. After reviewing criteria and discussing the risks and benefits with the Veteran, the pharmacist will initiate the appropriate dose of SGLT2i. Notification of the drug change will be sent by view alert to the patient's primary care provider. The pharmacist will schedule follow up labs 3 months after drug initiation and provide a phone number to Veterans to contact the pharmacist for future questions or to report adverse events. Labs at 3 months will be reviewed by the program's LPN. The Veteran will be contacted by letter with a summary of their lab results (sent by LPN) and reminded of our contact number. Any issues with labs will be flagged to the project PharmD to be addressed with the Veteran.

Implementation plan

The goal of the project is to start every eligible patient on an SGLT2 inhibitor. Currently in VISN23 over 8,000 patients are eligible for this program. Our initial project roll out is scheduled to have two PharmDs dedicated to the project. We anticipate each PharmD can initiate

approximately 1,000 patients on an SGLT2i in a year. As such, we can start 2,000 in the first year. There are no established criteria on the order in which to identify patient for participation (e.g., severity of disease). We are choosing to limit our implementation plan in the first year to those patients in Minneapolis and St. Cloud (about 2,000 patients). Of these, we will initially approach those whose social security number ends in an odd digit. In subsequent years, we will approach patients in the rest of VISN23 sites with odd ending SSNs for drug imitation. Once all of the odd SSN patients have been approached, we will then target those with an even SSN. The goal of the program is to target 100% of eligible patients.

The overall aim of this analysis plan is to determine if a targeted implementation plan with dedicated PharmD's improves adoption of SGLTi use among those with CKD compared to traditional VA care. If a targeted implementation plan is successful, future roll outs of beneficial interventions can follow a similar methodology.

Data collection and analysis

This quality improvement initiative will run for two years. Our primary outcome of interest is percent of patients that are initiated on SGLT2i therapy between the two groups (those who receive usual care vs. those who receive a letter and proactive pharmacist consultation). If adequate data is available, we would like to also evaluate if there was a difference in chronic kidney disease progression and cardiovascular outcomes. In addition, we plan to evaluate several implementation outcomes, such as reach (i.e., the proportion of eligible patients that receive the intervention), patient perception of starting this medication via qualitative interviews, and early-stage implementation barriers and facilitators gathered from the pharmacy service care team.

Documentation of non-research determination decision process

Using the criteria detailed in the VHA Office of Research Oversight Decision Chart for Determining VA Operations Activities That May Constitute Research (available at www.va.gov/oro/oropubs.asp), this quality improvement project is not research. quality improvement project is designed for internal VA purposes in support of the VA mission and will determine whether a pharmacy service program will improve SGLT2i treatment rates among Veterans with chronic kidney disease and diabetes. This QI project is not designed for the purpose of expanding the knowledge base of a scientific discipline or scholarly field of study and is not a clinical investigation as defined by the FDA. The findings of this QI project are designed to be used by and within the VA. This QI project is specifically designed to inform programmatic decisions by VISN 23 leadership (i.e., whether to roll out the initiative VISN-wide). This QI project is not funded or supported as research and is funded by VISN 23 clinical operations.

Is this project research?

Project title: SGLT2i to prevent cardiovascular disease and dialysis in patients with chronic kidney disease (CKD)

Primary contact for project: Areef Ishani

Email address: Areef.Ishani@va.gov Phone: 612-467-4431

Status at VA (e.g., MVAHCS staff, resident, trainee): Physician/Director

Supervisor: Larry Brown (VISN23 CMO)

IMPORTANT ➔Attach an abstract that describes the project purpose, methods, etc.

	YES	NO
A. Is the Operations Activity **designed** (and/or implemented) for **internal VA purposes in support of the VA mission(s)**? *(i.e., Is the project primarily intended to improve local processes of care and/or patient outcomes?)*	☑	☐
B. Are the activity's **findings designed to be used by and within VA** (or by entities responsible for overseeing VA)? *(i.e., Do you anticipate results from this project will be used to improve local processes of care?)*	☑	☐
➢ Is *either* question above answered **NO**? If so, **STOP HERE** and contact IRBMN@VA.GOV for further review.<br>➢ Are *both* questions above answered as **YES**? Continue to next section:		
1. Is the activity **designed** for the purpose of contributing to **generalizable knowledge**?	☐	☑
2. Is the activity **designed** for the purpose of expanding the **knowledge base** of a **scientific discipline** or scholarly field of study?	☐	☑
3. Is the activity funded or supported **as research**?	☐	☑
4. Is the activity a **clinical investigation** as defined under Food and Drug Administration (FDA) regulations?	☐	☑
5. Does the activity include **double-blind interventions**?	☐	☑
6. Does the activity include **placebo controls**?	☐	☑
7. Does the activity include **prospective patient-level randomization** to a clinical intervention **not tailored to individual patient benefit**?	☐	☑
8. Has the activity been s**upplemented or modified before, during, or after implementation** in order to produce information to **expand the knowledge base of a scientific discipline** or scholarly field of study or otherwise **contribute to generalizable knowledge**?	☐	☑
9. Has the purpose of the activity **changed** so that it is **now designed or intended** to expand the **knowledge base of a scientific discipline** or scholarly field of study or otherwise **contribute to generalizable knowledge**?	☐	☑
**To the best of my knowledge, the above is accurate.**<br>**Boxes 1 through 9 are checked NO, indicating this project is NOT research.**<br>Signature of person conducting project Ishani, Areef Digitally signed by Ishani, Areef Date: 2022.01.15 09:48:33 -06'00'	☑	
